# Research on Location Algorithm Based on Beacon Filtering Combining DV-Hop and Multidimensional Support Vector Regression

**DOI:** 10.3390/s21165335

**Published:** 2021-08-07

**Authors:** Dejing Zhang, Xiangcheng Zhang, Fengfeng Xie

**Affiliations:** School of Mechanical, Electrical & Information Engineering, Shandong University, Weihai 264209, China; zhangxc@mail.sdu.edu.cn (X.Z.); 201836542@mail.sdu.edu.cn (F.X.)

**Keywords:** wireless sensor network, DV-Hop, RSSI, MSVR, anisotropic networks

## Abstract

The DV-Hop algorithm is widely used because of its simplicity and low cost, but it has the disadvantage of a large positioning error. In recent years, although some improvement measures have been proposed, such as hop correction, distance-weighted correction, and improved coordinate solution, there is room for improvement in location accuracy, and the accuracy is affected in anisotropic networks. A location algorithm based on beacon filtering combining DV-Hop and multidimensional support vector regression (MSVR) is proposed in this paper. In the process of estimating the coordinates of unknown nodes, received signal strength indication (RSSI), MSVR, and weighted least squares method are combined. In addition, the verification error of beacon nodes is proposed, which can select the beacon nodes with smaller errors to reduce the location error. Simulation results show that in different distributions, the location accuracy of the proposed algorithm is at least 34% higher than that of the classical DV-Hop algorithm and at least 28% higher than that of the localization based on multidimensional support vector regression (LMSVR) algorithm. The proposed algorithm has the potential of application in small-scale anisotropic networks.

## 1. Introduction

The wireless sensor network (WSN), as a system-level project, is divided into multiple fields for research [1]. Because many location-aware protocols and applications need to obtain location information, determining the location of unknown nodes, as a critical technology, has always been a research hotspot [2]. Location algorithm in WSN can be divided into distance-based location algorithm and distance-free location algorithm. DV-Hop algorithm, as a classical distance-free location algorithm, is widely used because of its low network communication overhead and energy consumption. However, DV-Hop algorithm produces error accumulation in calculating minimum hop count, average hop distance, and in estimating unknown node coordinates, resulting in low overall accuracy [3]. Therefore, reducing the cumulative error of the DV-Hop positioning algorithm is important for accurate positioning.

In order to solve this problem, many studies have proposed improved methods in all stages of the positioning process. In the stage of obtaining the hop count between nodes, Qi Q et al. [4] corrected the hop count by a correction parameter; Pingzhang Gou et al. [5] subdivided the hop count with a double communication radius to estimate a more accurate average hop distance. In addition, the improvement based on received signal strength indication (RSSI) is often used in the research. RSSI is a method used to obtain distance information from the received signal strength according to the characteristics of wireless signal attenuation with distance. Because of its low cost and ease of use, it is a common method combined with distance in DV-Hop improvement. Dalong Xue [6] used RSSI to subdivide the first hop to obtain more accurate hop information. In the stage of obtaining the distance from the unknown node to the beacon node, the improved method is mainly based on different forms of weighted correction of the average hop distance [7,8,9]. Finally, in the stage of estimating the coordinates of unknown nodes, the traditional DV-Hop algorithm uses the least squares method to estimate the coordinates of unknown nodes. An error of the estimated distance between the beacon node and the unknown node easily affects the estimation result, which leads to a large deviation between the estimated position of the node and the actual position. At present, the main improved methods are weighted least squares method [10], two-dimensional hyperbolic algorithm [11], differential evolution algorithm [12], simulated annealing algorithm [13], and particle swarm optimization algorithm [14]. In addition to the weighted least squares method, simulated annealing algorithm is one of the most popular improved methods. Moreover, in recent years, some scholars have proposed to combine machine learning theory with location algorithm. LMSVR (localization based on multidimensional support vector regression), proposed by Jaehun Lee [15], trains the multidimensional support vector regression (MSVR) model by a new training method. By using this method, a two-dimensional MSVR model can be obtained, and the coordinates of unknown nodes can be estimated by the hop information between nodes. Compared with the traditional DV-Hop, this method has a certain improvement, but the improvement effect of the algorithm is not obvious. Moreover, by directly using hops to estimate coordinates, the method cannot make full use of the location information of nodes in the network. In addition, the research on location algorithms should also consider the attributes of the network. According to the network attributes, wireless sensor networks can be divided into isotropic networks and anisotropic networks. An isotropic network is a network whose physical properties are consistent in all directions, and an anisotropic network is a network in which the physical properties of the network change with the direction of measurement. In recent years, many scholars have proposed improved methods for the application of MSVR in location algorithms. JiandongYao et al. [16] viewed the process of location estimation as a regression prediction and predicted node coordinates according to the distance information obtained by hop mapping. This method improved the location accuracy, but constructing the mapping relationship between hop value and distance is not applicable in anisotropic networks. Moreover, NiharikaAnand et al. [17] proposed a new MSVR training method, and Paria M. et al. [18] proposed a different kernel function. Although these methods have improved the MSVR, the improvement of location accuracy is not obvious, so there is still room for improvement.

To solve this problem, this paper proposes a MSVR-DV-Hop algorithm based on beacon filtering. Firstly, RSSI is introduced to subdivide the hop count, and then MSVR is extended to be N-dimensional to obtain the distance between unknown nodes and beacon nodes. Finally, beacon node verification errors are introduced to filter beacon nodes with high reliability, and the coordinates of unknown nodes are estimated by weighted least squares method. The error caused by the MSVR model and the influence of the error in coordinate estimation are reduced. At present, most of the improved algorithms consider isotropic networks, and there are few experiments on anisotropic networks [19]. Therefore, this paper also studies the applicability of the algorithm in anisotropic networks.

The rest of this paper is organized as follows: in Section 2, the algorithm background of this paper is explained, and the existing problems of each algorithm are analyzed. In Section 3, the basic flow of the algorithm proposed in this paper is introduced. In Section 4, the proposed algorithm is simulated with several other representative algorithms, and its location performance is analyzed. The conclusion of this paper is given in Section 5.

## 2. Classic DV-Hop Error Analysis and Localization Based on Multidimensional Support Vector Regression Model

### 2.1. Classical DV-Hop Error Analysis

The error of the classical DV-Hop algorithm [20] mainly comes from three aspects.

**Figure 1:**
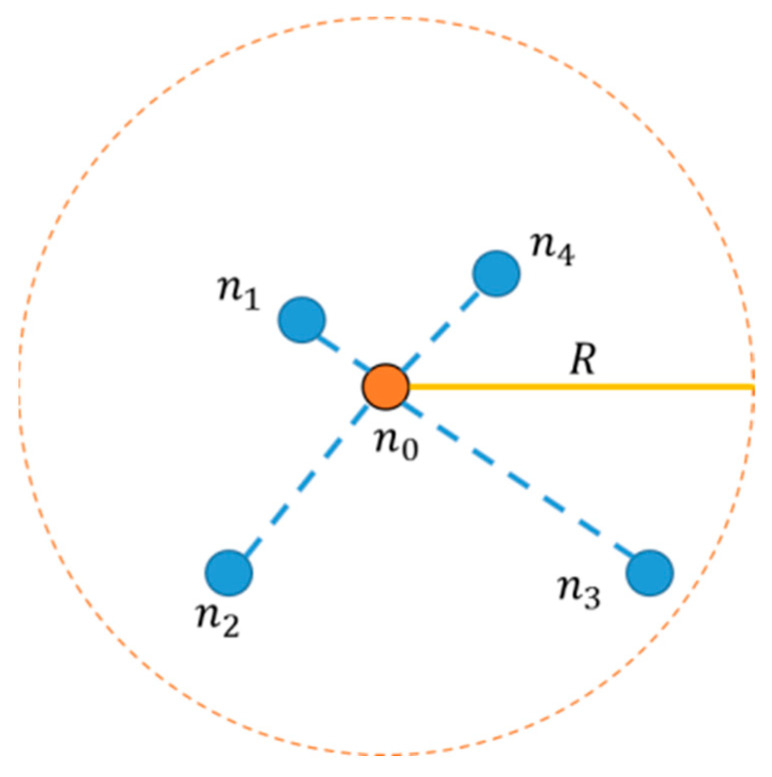
Schematic diagram of the error of hop value.

(1) The error of hop value occurs due to an uneven distribution of nodes, as shown in Figure 1.
  Suppose there are four neighboring nodes, $n_{1},n_{2},n_{3},$ and $n_{4}$, within the communication radius R of node $n_{0}$, and the distance between them is $d_{0.3}>d_{0.2}>d_{0.4}>d_{0.1}$. Messages of $n_{0}$ can reach the other four nodes in one hop, so all four neighboring nodes have the hop value of 1. In the calculation process of the algorithm, the distance between nodes is a product of the hop values and the average hop distance $D_{avrage}$, that is, $d_{0.3}=d_{0.2}=d_{0.4}=d_{0.1}=1\times D_{avrage}$, which is obviously inconsistent with the reality. There is a significant difference in the distance from $n_{0}$ to the four neighboring nodes, and this part of the error will affect the coordinate estimation.
(2) When calculating the distance between the unknown node and the beacon node, the average hop distance to the nearest beacon node is used. Because the nodes connecting two beacon nodes cannot be uniformly distributed in a straight line, the average hop distance calculated is often less than the actual value, resulting in the location error of unknown nodes.
(3) In the stage of node coordinate estimation, because there are inevitably some errors in the distance estimate in the second stage of the algorithm, these errors will accumulate when solving the coordinate equations, resulting in a larger error between the final result and the actual coordinate.

### 2.2. Localization Based on Multidimensional Support Vector Regression Model

LMSVR constructs a regression model by using the training data pair ($h_{i}$,$\;I_{i}$) of beacon nodes, and the regression model outputs the estimated coordinates of unknown nodes, where $h_{i}$ is the hop number vector from node to beacon node, and $I_{i}$ is the node coordinate. When the regression model is constructed according to the training dataset, the multidimensional regression function is

(1) $$f\left(h_{i}\right)=W\varphi \left(h_{i}\right)+b=\left[\begin{array}{c} w_{x}^{T}\\ w_{y}^{T} \end{array}\right]\varphi \left(h_{i}\right)+\left[\begin{array}{c} b_{x}\\ b_{y} \end{array}\right]$$

where W is the weight matrix, b is the two-dimensional regression deviation vector, and φ is the nonlinear function.

The ε-insensitive quadratic loss function $L_{\varepsilon }\left(z\right)$ and transition variable α can be defined as follows:

(2) $$L_{\varepsilon }\left(z\right)=\{\begin{array}{l} 0,\;z\leq \varepsilon \\ {\left(z-\varepsilon \right)}^{2},\;otherwise \end{array}$$

(3) $$\alpha_{i}=-\frac{C}{2}\frac{\partial }{\partial w}L_{\varepsilon }\left(\|I_{i}-f\left(h_{i}\right)\|\right)$$

The MSVR training process can be equivalent to an optimization problem:

(4) $$\begin{array}{l} \;\;\mathrm{Minimize}\alpha ,\xi \\ \lambda \left(\overset{M}{\underset{i,j=1}{\sum }}\alpha_{i,x}\alpha_{j,x}k\left(h_{i},h_{j}\right)+\overset{M}{\underset{i,j=1}{\sum }}\alpha_{i,y}\alpha_{j,y}k\left(h_{i},h_{j}\right)\right)+\overset{M}{\underset{i=1}{\sum }}\xi_{i}^{2}\\ \;\;\;\;\;\;\;\;s.t.\;\|I_{i}-\overset{M}{\underset{j=1}{\sum }}\alpha_{j}k\left(p_{j},p_{i}\right)-b\|\leq \varepsilon +\xi_{i},\xi_{i}\geq 0 \end{array}$$

where I is the coordinate vector of the beacon node, C is the soft margin parameter, $\xi_{i}$ are the slack variables, and λ=1/C, $k\left(h_{i},h_{j}\right)$ is the kernel function, which is a Gaussian function in this paper.

The unknown nodes can predict their own coordinates using the α, ξ obtained from the optimization problem. It is worth noting that LMSVR is an algorithm based on machine learning. Although this algorithm can improve the accuracy and stability of coordinate estimation to some extent, it also has some common problems that are easy to encounter in machine learning. For example, when the number of beacon nodes is small, the location error will obviously increase due to the lack of training samples and the inaccuracy of the training MSVR model, and more errors in the estimated distance between nodes are generated, thus the accuracy of positioning is affected. On the other hand, the influence of a single sample error on the whole is more obvious, which is also one of the reasons for the increase of error. To solve these problems, a MSVR-DV-Hop algorithm based on beacon filtering is proposed in this paper.

## 3. Algorithm Process

The proposed algorithm in this paper consists of three steps. Firstly, the hop count is obtained by RSSI correction; then, the distance estimation and beacon filtering are carried out based on MSVR, and finally, the coordinates of unknown nodes are calculated by the distance-weighted least squares method. The specific steps of the algorithm are as follows.

### 3.1. First Hop Grading

In this paper, based on the classical DV-Hop algorithm for obtaining hop count, the first hop is graded with RSSI. We estimate the relationship between d and R on the basis of the measured RSSI value. Then, the first hop is divided into n levels. The grading method is as follows:

(5) $$h=\{\begin{array}{l} \frac{1}{n},\;A-10k_{0}lg\left(\frac{R}{n}\right)\leq P_{r}\left(d\right)<0\\ \frac{i}{n},\;A-10k_{0}lg\left(\frac{iR}{n}\right)\leq P_{r}\left(d\right)<A-10k_{0}lg\left(\frac{\left(i-1\right)R}{n}\right)\\ 1,\;A-10k_{0}lg\left(R\right)\leq P_{r}\left(d\right)<A-10k_{0}lg\left(\frac{\left(n-1\right)R}{n}\right) \end{array}$$

where d is the distance between the two nodes, R is the communication radius, A is the signal strength received when the distance between nodes is 1 m, and $k_{0}$ is the propagation factor of the wireless signal. $k_{0}$ is an empirical value, which is related to the hardware node and the environment. It is usually set between 2 and 6. Although there is an error of RSSI in the actual environment, this method does not need an accurate RSSI value. It only needs to get a fuzzy value to grade the hop, so as to reduce the error of hop value.

### 3.2. Distance Prediction

In the distance calculation stage, the sink node trains MSVR with the hops information after grading (obtained in Section 3.1) and the distance between each beacon node. Then, the parameters obtained by the optimal model are sent to each unknown node, and the unknown node predicts the distance to the beacon node by combining the number of hops from itself to each beacon node.

The training model of N-dimensional MSVR is

(6) $$f_{MSVR}\left(p_{i}\right)=\omega^{T}\varphi \left(p_{i}\right)+b=\left[\begin{array}{c} \omega_{1}^{T}\\ \vdots \\ \omega_{n}^{T} \end{array}\right]\varphi \left(p_{i}\right)+\left[\begin{array}{c} b_{1}\\ \vdots \\ b_{n} \end{array}\right]$$

where n is the number of beacon nodes.

### 3.3. Verification Error of Beacon Node

In the distance calculation stage, a verification error of beacon nodes is designed to correct the positioning deviation caused by a small number of beacon nodes with high error. In the sink node, the distance vector between the beacon node i and other beacon nodes is $\left(d_{1},d_{2},\ldots ,d_{n-1}\right)$. The MSVR model is trained with the hop count and distance information of beacon nodes except beacon i, and then the distance vector $\left(d_{1}^{pred},d_{2}^{pred},\ldots ,d_{n-1}^{pred}\right)$ from beacon i to other beacon nodes is predicted. The verification error is calculated by comparing the actual distance vector with the predicted distance vector.

(7) $$e_{i}=\overset{n-1}{\underset{j=1}{\sum }}\left|d_{j}-d_{j}^{pred}\right|$$

### 3.4. Estimation of Unknown Node Coordinates

After the unknown node receives the verification error of beacon nodes and calculates the estimated distance to each beacon, the credibility of each beacon node is evaluated according to the verification error of each beacon node and the distance to these beacons. The evaluating rules are shown in Table 1. When the distances to the unknown node $d_{i}$ and the verification error $e_{i}$ of beacon node i are both large, the predicted distance to the beacon node is generally quite different from the actual distance. Therefore, beacon i is considered to be unreliable and unavailable to be selected as a reference. Otherwise, if the beacon is selected as a reference when solving the least squares equation, the positioning accuracy will be affected.

After filtering unavailable beacon nodes, the coordinates are calculated by the weighted least squares method with a weight of 1/d.

### 3.5. Algorithm Analysis

The MSVR-DV-Hop algorithm based on beacon filtering proposed in this paper introduces RSSI grading to obtain more accurate hop number information, which provides more data in line with the actual position for the training of MSVR model. Therefore, the trained MSVR model will be more accurate. In the distance calculation stage, the multidimensional MSVR is used to obtain the distance between the unknown node and the beacon node. Moreover, the process of estimating node coordinates by hop number is divided into two parts, so the error caused by the MSVR model is further modified to increase the accuracy of the algorithm. In the coordinate estimation stage, the beacon node verification error is designed according to the mutual verification between beacon nodes. The unknown node eliminates the beacon node with high error according to the verification error and the distance to the beacon node. Finally, the weighted least squares method is used to calculate the estimated coordinates of the unknown node. By filtering the beacon nodes, the influence of the beacon node error can be reduced in solving the coordinate equation, which the beacon nodes with large error cannot participate in.

Since the algorithm complexity of MSVR is related to the dimension of input vector $d_{L}$ and the number of training sample points l [21], the expansion of two-dimensional MSVR to N-dimensions will inevitably lead to the increase of computational complexity. Although algorithm complexity can be alleviated to some extent by filtering which can reduce the number of beacon nodes as training samples, the overall computational complexity of the algorithm still increases.

Above all, although the proposed algorithm increases the computation complexity when expanding the MSVR model and calculating the verification error, it can significantly reduce the location error, which is still acceptable in some scenarios. Moreover, the proposed algorithm does not rely on the average hop distance when obtaining the distance between nodes, so the positioning accuracy will not decrease significantly in an anisotropic scenario where the distance is not necessarily proportional to the number of hops.

## 4. Simulation and Analysis

**Figure 2:**
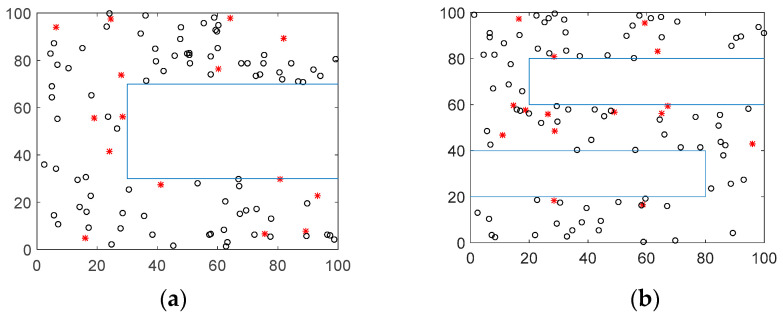

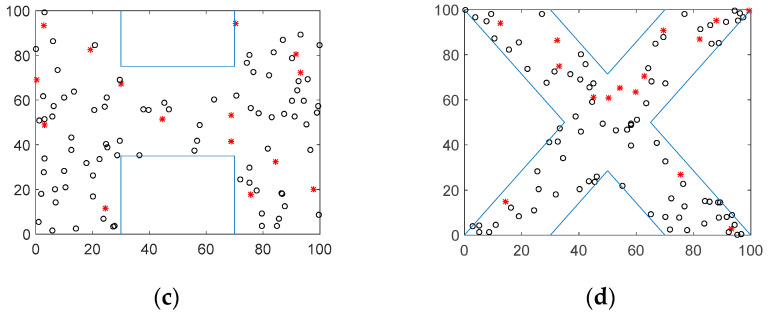
Anisotropic networks in simulation. (**a**) C-shape network. (**b**) S-shape network. (**c**) H-shape network. (**d**) X-shape network.

In order to evaluate the performance of the algorithm proposed in this paper, the topology distribution with different network parameters was simulated in MATLAB R2015b. In the simulation, both the isotropic network and the anisotropic network were considered. Specifically, four types of the representative anisotropic networks were adopted. Since the evaluation in anisotropic networks is more rigorous than in other networks, the result of simulation can be more reliable. The nodes of the isotropic network were randomly distributed in a 100 m × 100 m square area, and the four typical topologies of anisotropic networks are shown in Figure 2. The initial parameters were set as follows: total number of nodes: 100, proportion of beacon nodes: 15%, and communication radius: 50 m. The simulation parameters are shown in detail in Table 2. Compared with the isotropic network, the anisotropic network is more practical. However, as the correlation between hop numbers and distances between nodes decreases, it has a greater impact on the accuracy of the localization algorithm. Specific analysis will be mentioned later.

The proposed algorithm was compared with several common improved algorithms. The classical DV-Hop algorithm, LMSVR algorithm [14], and DV-Hop [22] based on simulated annealing algorithm were selected and compared with the proposed algorithm before and after beacon filtering. In the simulation results, SVR-wlsqu is the proposed algorithm before beacon filtering. The location error adopts the average location error, as shown in Equation (8).

(8) $$ALE=\frac{\sum_{i=1}^{n}\sqrt{{\left({\hat{x}}_{i}-x_{i}\right)}^{2}+{\left({\hat{y}}_{i}-y_{i}\right)}^{2}}}{nR}$$

where $\left(\hat{x},\hat{y}\right)$ is the estimated coordinate, n is the number of nodes, and R is the communication radius.

**Figure 3:**
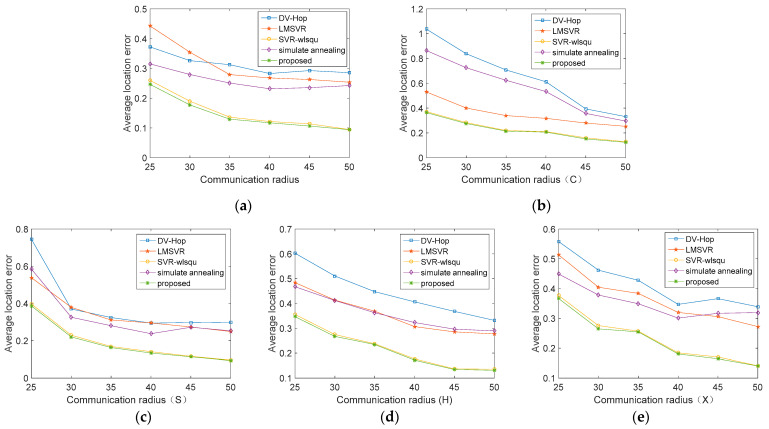
Comparison of the average location error under various communication radius distances. (**a**) isotropic networks. (**b**) C-shape network. (**c**) S-shape network. (**d**) H-shape network. (**e**) X-shape network.

Figure 3 shows the effect of each algorithm under different topology distributions as the communication radius R increases from 25 m to 50 m. It can be seen that the location error of the proposed algorithm is significantly lower than that of other algorithms in different topology distributions, and the location effect is obviously improved with the increase of communication radius. Compared with the isotropic network, the error of each algorithm in the anisotropic network increases in varying degrees. This is due to the fact that there is an area in which the nodes cannot be distributed in the anisotropic network, so that there is no longer a linear relationship between the hop count and the distance of the nodes. This phenomenon will be alleviated to a certain extent as the communication radius increases. In all subgraphs in Figure 3, the location error of DV-Hop and simulated annealing DV-Hop in the anisotropic network increases obviously; however, in contrast, LMSVR and the proposed algorithm have a small increase in location error, which shows that DV-Hop based on MSVR can better extract and make use of hidden network information. Therefore, it has more advantages in dealing with the anisotropic networks.

**Figure 4:**
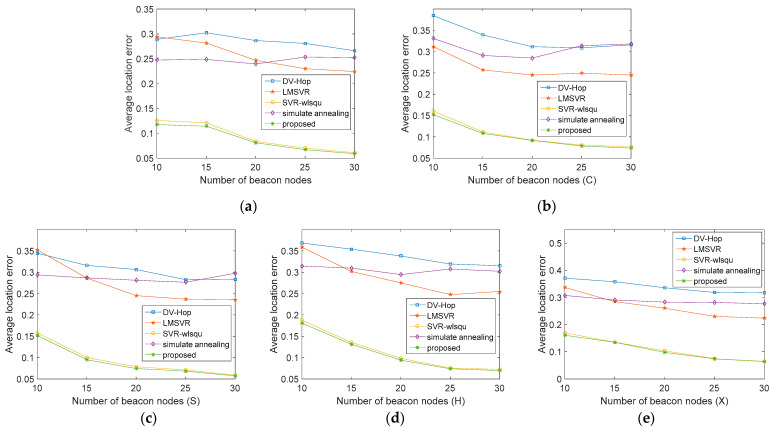
Comparison of the average location error under various numbers of beacon nodes. (**a**) isotropic networks. (**b**) C-shape network. (**c**) S-shape network. (**d**) H-shape network. (**e**) X-shape network.

Figure 4 shows the simulation results obtained as the proportion of beacon nodes increases from 10% to 30%. The proposed algorithm performs better than other algorithms in various environments of beacon nodes, and the effect is greater in the environment with a high proportion of beacon nodes. This is because the trained MSVR model is more accurate when the proportion of beacon nodes is high. Moreover, more nodes can be used in the coordinate estimation stage, and the network information is also more detailed. Considering the results of this algorithm in different regions, we can also see that the algorithm is less affected by the network topology and can still achieve high accuracy in complex cases.

**Figure 5:**
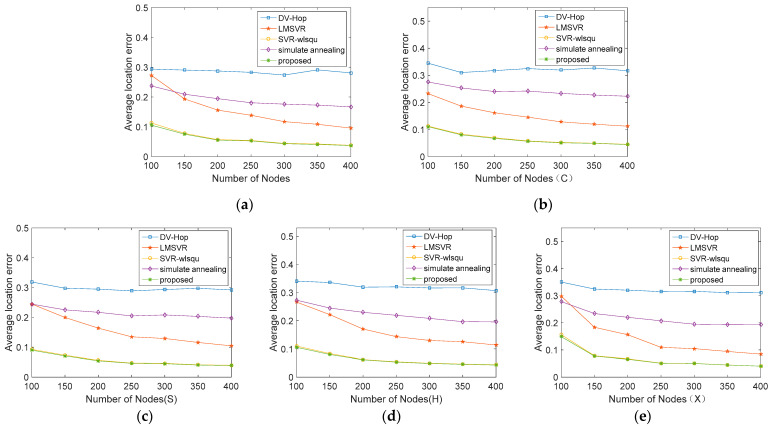
Comparison of the average location error under various total number of nodes. (**a**) isotropic networks. (**b**) C-shape network. (**c**) S-shape network. (**d**) H-shape network. (**e**) X-shape network.

Figure 5 shows a comparison of the results of the algorithms as the total number of nodes increases from 100 to 400. The algorithm proposed in this paper performs better than other algorithms in all environments, and the results are less affected by the total number of nodes. The error only decreases obviously when the total number increases from 100 to 200. In contrast, the positioning accuracy of LMSVR decreases gradually with the increase of the total number of nodes and the number of beacon nodes, but it does not reach the accuracy of the algorithm proposed in this paper. By comparison, the algorithm proposed in this paper still shows high accuracy in small-scale anisotropic networks, and is not easily affected by the size of the network.

In each distribution area, the average location errors of different environments of communication radius, number of beacons, and total number of nodes are averaged to evaluate the comprehensive performance of the algorithm. This value reflects the sensitivity of the algorithm to the network parameters in each distribution region. It can be seen from Table 3 that the adaptability of the proposed algorithm for network parameters is the highest.

To sum up, the algorithm proposed in this paper performs better than classical DV-Hop, simulated annealing algorithm, and LMSVR in different environments. In addition, the location error of the algorithm after beacon filtering is lower than that before beacon filtering, and this phenomenon is more prominent in the environment where the total number of nodes is small and the proportion of beacon nodes is low. This is because when there are few beacon nodes, a small number of beacon nodes with large errors participate more in the process of coordinate estimation. However, after the beacons are screened by verification errors, the impact of these nodes on the estimated results can be effectively avoided. After analysis, the algorithm proposed in this paper shows good performance in the scenario with a small number of beacon nodes.

## 5. Conclusion

In order to solve the problem of large location error of the traditional DV-Hop algorithm, this work combined the LMSVR algorithm with the DV-Hop algorithm and proposes a MSVR-DV-Hop algorithm based on beacon filtering. The hop count is graded by RSSI, and the distance from the unknown node to the beacon node is obtained by N-dimensional MSVR. Finally, the weighted least squares method is used to solve the equation estimation. The experimental results show that the algorithm proposed in this paper reduces the location error and has high positioning accuracy. The algorithm also improves the problem of an increased error of LMSVR when the number of beacon nodes is small. Moreover, the performance of this algorithm will not be significantly reduced in anisotropic networks, and it has high positioning accuracy and stability. In addition, the increase in complexity due to MSVR will affect the lifetime of the sink nodes. Although the algorithm is acceptable in some networks, future research will focus on how to reduce its energy consumption.

## Figures and Tables

**Table 1:** Beacon node filter table.

	Distance	≤*e_mean_*	>*e_mean_*
Validation Error			
≤*d_mean_*		Available	Available
>*d_mean_*		Available	Unavailable

**Table 2:** The simulation parameters.

Parameter	Value
The area size	100 × 100 m
Communication radius	25–50 m
Beacon node ratio	10–30%
Total number of nodes	100–400
Hop thinning level	3

**Table 3:** Comprehensive performance of algorithms in different networks.

		DV-Hop	Simulated Annealing	LMSVR	Proposed
isotropic	*R*	0.3732	0.3130	0.3266	0.1665
	$N_{\mathrm{Anchor}}$	0.2933	0.2549	0.2574	0.0904
	$N_{\mathrm{Node}}$	0.2856	0.1909	0.1545	0.0585
C-shaped	*R*	0.6532	0.5664	0.3534	0.2225
	$N_{\mathrm{Anchor}}$	0.3263	0.2922	0.2441	0.0933
	$N_{\mathrm{Node}}$	0.3228	0.2420	0.1551	0.0654
S-shaped	*R*	0.3887	0.3257	0.3410	0.1851
	$N_{\mathrm{Anchor}}$	0.3118	0.2899	0.2730	0.0924
	$N_{\mathrm{Node}}$	0.2977	0.2143	0.1560	0.0555
H-shaped	*R*	0.4446	0.3586	0.3561	0.2144
	$N_{\mathrm{Anchor}}$	0.3370	0.3036	0.2805	0.1072
	$N_{\mathrm{Node}}$	0.3225	0.2274	0.1673	0.0625
X-shaped	*R*	0.4166	0.3523	0.3666	0.2286
	$N_{\mathrm{Anchor}}$	0.3354	0.2881	0.2682	0.1038
	$N_{\mathrm{Node}}$	0.3230	0.2214	0.1576	0.0729

## Data Availability

Not applicable.
